# IL-8 promotes inflammatory mediators and stimulates activation of p38 MAPK/ERK-NF-κB pathway and reduction of JNK in HNSCC

**DOI:** 10.18632/oncotarget.16914

**Published:** 2017-04-07

**Authors:** Leong-Perng Chan, Cheng Liu, Feng-Yu Chiang, Ling-Feng Wang, Ka-Wo Lee, Wan-Ting Chen, Po-Lin Kuo, Chia-Hua Liang

**Affiliations:** ^1^ Graduate Institute of Clinical Medicine, Kaohsiung Medical University, Kaohsiung, Taiwan; ^2^ Department of Otolaryngology-Head and Neck Surgery, Kaohsiung Medical University Hospital, Kaohsiung Medical University, Kaohsiung, Taiwan; ^3^ Division of Plastic Surgery & HBOT Center, Chi Mei Medical Center, Tainan, Taiwan; ^4^ Department of Electrical Engineering, Southern Taiwan University of Science & Technology, Tainan, Taiwan; ^5^ Department of Cosmetic Science and Institute of Cosmetic Science, Chia Nan University of Pharmacy and Science, Tainan, Taiwan; ^6^ Institute of Medical Science and Technology, National Sun Yat-Sen University, Kaohsiung, Taiwan

**Keywords:** HNSCC, IL-8, inflammation, MAPK

## Abstract

This investigation identifies interleukin 8 (IL-8) as the main inflammatory mediator in head and neck squamous cell carcinoma (HNSCC). The expressions of chemokines of IL-8, IL-1β and IL-6 and the cytokines of tumor necrosis factor-α (TNF-α) were higher in HNSCC patient tissues than in non-cancerous matched tissues (NCMT) whereas the expression of IL-10 was lower. IL-8 is most highly expressed in the tissues of patients with HNSCC. Treatment of HNSCC cells with IL-8 increased the secretion of IL-1β, IL-6 and TNF-α and reduced IL-10 expression; the increase in the expression of IL-1β was particularly considerable. IL-8 silencing by siRNA reduced IL-1β expression in HNSCC cells, suggesting that IL-8 as a main inflammatory mediator improved IL-1β expression in HNSCC. The expressions of p-p38 mitogen-activated protein kinases (MAPK) and p-extracellular signal regulated kinase (p-ERK) were higher and that of p-c-Jun-NH2-terminal kinase (p-JNK) was lower in HNSCC patient tissues than in NCMT. IL-8 treatment induced p-p38 MAPK and p-ERK expression, but reduced p-JNK expressions in HNSCC cells. IL-8 siRNA suppressed p38 MAPK and ERK but increased JNK expression in HNSCC cells. Exposure of SCC25 cells to IL-8, increased the expressions of p-IκB-α and nuclear factor (NF)-κB, suggesting that IL-8 regulates inflammatory response by modulating the MAPK and NF-κB pathway in HNSCC cells. IL-8 promotes the migration of SCC25 cells and increases matrix metalloproteinase-2 (MMP-2) and MMP-9 expressions. These results reveal that IL-8 is the major stimulus of inflammatory mediation in HNSCC progression and migration by activating the p38 MAPK/ERK-NF-κB pathway and reducing JNK.

## INTRODUCTION

HNSCC is the sixth most common cancer globally, affecting over 400,000 patients and causing over 200,000 deaths annually [1]. Over the last few decades, substantial progress has been made in the diagnosis and treatment of HNSCC. However, HNSCC is still associated with a high rate of mortality, and prognosis for this tumor is poor, even with treatment that is considered potentially curative [2]. The fact that an improvement in the survival of patients with HNSCC depends on improving our understanding of tumorigenesis, metastasis and recurrence is becoming increasingly evident [3]. Molecular changes and mechanisms that regulate the development and progression of HNSCC remain unclear.

Tumor cells have been suggested to produce IL-8, which is a member of the C-X-C chemokine family, as an autocrine growth factor, which promotes tumor growth, tissue invasion and metastatic spread [4, 5]. Studies have revealed that highly metastatic solid tumors, such as oral, prostate, breast, melanoma and ovarian cancer, constitutively express IL-8 [6]. The biological action of IL-8 is mediated its binding to its receptors, CXCR1 (IL-8RA) and CXCR2 (IL-8RB), which are members of the seven transmembrane G-protein-coupled receptor (GPCR) family [7]. IL-8 has an important role in inflammation, tumor progression and angiogenesis in various tumors [8], but the co-operative functions of inflammatory mediators in HNSCC have not been clearly elucidated. Therefore, this investigation elucidates the co-operative effects of IL-8 on HNSCC progression, especially in relation to the interaction between inflammatory mediators and the microenvironment of the tumor.

Numerous studies of inflammatory diseases have addressed the importance of MAPKs in cell biology. MAPKs include p38, ERK, and JNK [9]. The involvement of MAPKs in cells of several types following receptor activation by inflammatory mediators such as chemotactic factors [IL-8, monocyte chemoattractant protein 1 (MCP-1), *N*-formyl-methionyl-leucyl-phenylalanine (fMLP), platelet-activating factor and complement factor 5a (C5a)], cytokines [IL-1β, TNF-α and lipopolysaccharide (LPS)] and growth factors [granulocyte-macrophage colony-stimulating factor (GM-CSF) and transforming growth factor β (TGF-β) generates cellular responses, such as the production, adhesion, chemotaxis, and degranulation of IL-8, as well as oxidative bursting [10]. IL-8 signaling has been demonstrated to induce the activation of this classic MAPK signaling cascade, with downstream phosphorylation of ERK in cancer cells, including prostate, ovarian and lung cancer cells [11]. The production of IL-8 has been demonstrated to depend on ERK activation, but the role of JNK in the regulation of IL-8 in HNSCC has rarely been investigated [12].

This study investigates 1) IL-8 expression in surgical specimens of HNSCC and its relationship with clinicopathological factors, 2) the co-operative effects of IL-8 and inflammatory mediators in HNSCC progression; 3) the relationship between the MAPK pathway and the expression of IL-8, and 4) the question of whether the silencing of IL-8 by siRNA affects inflammatory mediators and p38 MAPK, JNK and ERK pathways in HNSCC. The results suggest a relationship between the expression of IL-8 with HNSCC development and that the MAPK pathway is crucial in HNSCC cell growth.

## RESULTS

### Microarray, inflammatory mediators

**Figure 1:**
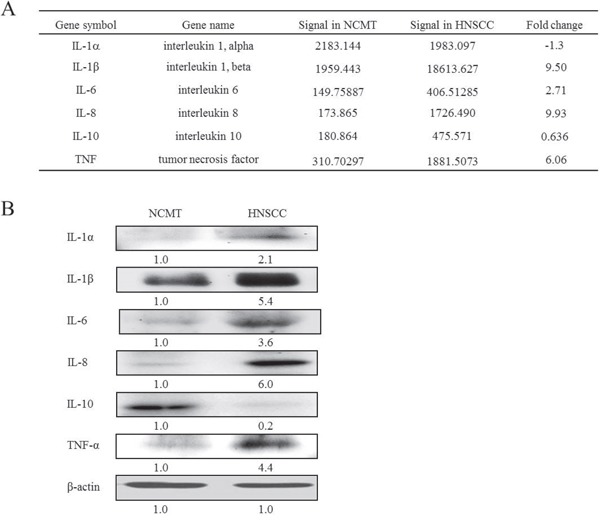
Expression of inflammatory mediators in NCMT and HNSCC of human tissue **(A)** A microarray analysis to evaluate expressions and the fold-change thresholds of IL-1α, IL-1β, IL-6, IL-8, IL-10 and TNF in NCMT and HNSCC of human tissue (patient no. 2). **(B)** IL-1α, IL-1β, IL-6, IL-8, IL-10 and TNF-α expressions in NCMT and HNSCC of human tissue (patient no. 3) were analyzed by western blotting. β-actin was used as internal control for sample loading.

Microarray technology has demonstrated the critical molecular and biological characteristics. Comprehensive analyses of the gene-expression profiles of hepatocellular carcinomas have taken into account various clinical characteristics, including the differences between tumor and non-tumorous tissues [13]. A microarray analysis was performed to determine the expression of the genes of inflammatory mediators in NCMT and HNSCC in human tissue (patient no. 2). The genes of several inflammatory mediators were found to be associated with the up-regulation of chemokines and cytokines in HNSCC relative to NCMT. The expressions of chemokines IL-8, IL-1β and IL-6 and cytokines TNF-α in HNSCC were significantly up-regulated (> 2-fold) relative to those in NCMT, and the up-regulation of IL-8 was particularly considerable. The increases were by factors of 9.93 for IL-8, 9.5 for IL-1β, 2.71 for IL-6, and 6.06 for TNF (Figure 1A).

First, microarray chip analysis was performed to screen gene expression, and the results were confirmed in other experiments, such as by using PCR. To identify the inflammatory mediators that support IL-8 expression in HNSCC, as established by the microarray investigation, the levels of IL-1α, IL-1β, IL-6, IL-8, IL-10 and TNF-α in HNSCC and NCMT tissues were obtained by western blotting. Incubation with an antibody against β-actin revealed an equal loading of protein. IL-1α, IL-1β, IL-6, IL-8 and TNF-α levels were higher (2.1, 5.4, 3.6, 6.0 and 4.4-fold, respectively) in HNSCC specimens than in NCMT, whereas the IL-10 level was 0.2-fold lower (Figure 1B). Although the array analysis revealed that the gene expression of IL-1α in HNSCC patient tissue was lower than in NCMT, subsequent western blot analysis revealed that the protein expression was slightly higher in the former. Therefore, the expression of IL-8 in HNSCC was higher than in NCMT, and the other inflammatory mediators exhibited lower expression. These results confirm that IL-8 is a major biomarker in the detection of HNSCC by microarray analysis (Figure 1A).

### IL-8 and inflammatory mediators in HNSCC

**Figure 2:**
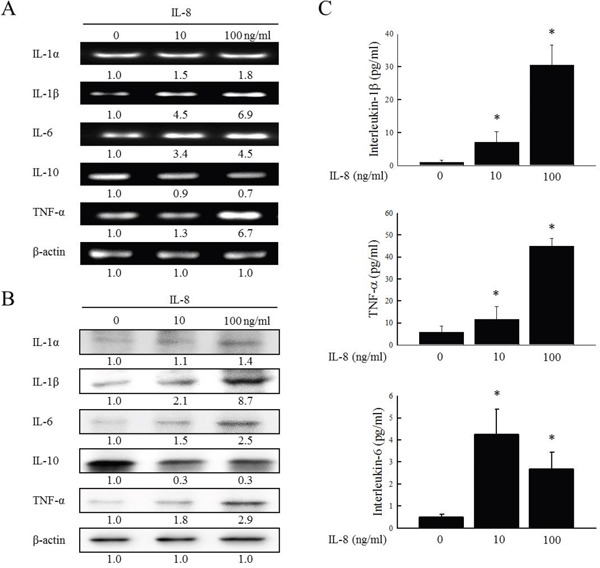
IL-8 and inflammatory mediators in HNSCC cells SCC25 were stimulated using IL-8 (10 and 100 ng/ml) for 72 h, and gene and protein expressions of IL-1α, IL-1β, IL-6, IL-10 and TNF-α were evaluated using RT-PCR **(A)** and western blotting **(B).** β-actin was used as internal controls for sample loading. **(C)** SCC25 cells were stimulated using IL-8 (10 and 100 ng/ml) for 72 h; culture media were then collected and tconcentrations of IL-1β, TNF-α and IL-6 were measured using an BD OptEIA ELISA kit. Each value is presented as mean ± SD across three experiments. *p<0.05 implies a significant difference from control cells.

To determine whether IL-8 is the dominant stimulus of inflammatory mediators and to examine the co-operative effects of inflammatory mediators in HNSCC, following the incubation of SCC25 cells with IL-8 (10 and 100 ng/ml) for 72 h, the gene and protein expressions of IL-1α, IL-1β, IL-6, IL-8, IL-10 and TNF-α were determined by RT-PCR and western blotting. As shown in Figure 2A, the up-expression of IL-1α, IL-1β, IL-6 and TNF-α and the down-expression of IL-10 genes were observed by RT-PCR after the exposure of IL-8 to SCC25 cells. Treatment with IL-8 (100 ng/ml) increased IL-1α, IL-1β, IL-6 and TNF-α mRNA levels 1.8, 6.9, 4.5 and 6.7-fold, respectively, and reduced the IL-10 level to 0.7-times its original value. As shown in Figure 2B, western blotting revealed that the treatment of SCC25 cells with IL-8 caused the up-expression of IL-1α, IL-1β, IL-6 and TNF-α protein and the down-expression of IL-10. Treating SCC25 with IL-8 (100 ng/ml) increased IL-1α, IL-1β, IL-6 and TNF-α protein levels 1.4, 8.7, 2.5 and 2.9-fold, respectively, and reduced the IL-10 protein level to 0.3 times its original value. The changes the expression of IL1-β, TNF-α, and IL-6 are more significant than that of IL-1α. These analytical results suggest that IL-1β, TNF-α and IL-6 were the main inflammatory mediators that were up-regulated in response to IL-8, and that the IL-1β expression was substantially up-regulated.

**Figure 3:**
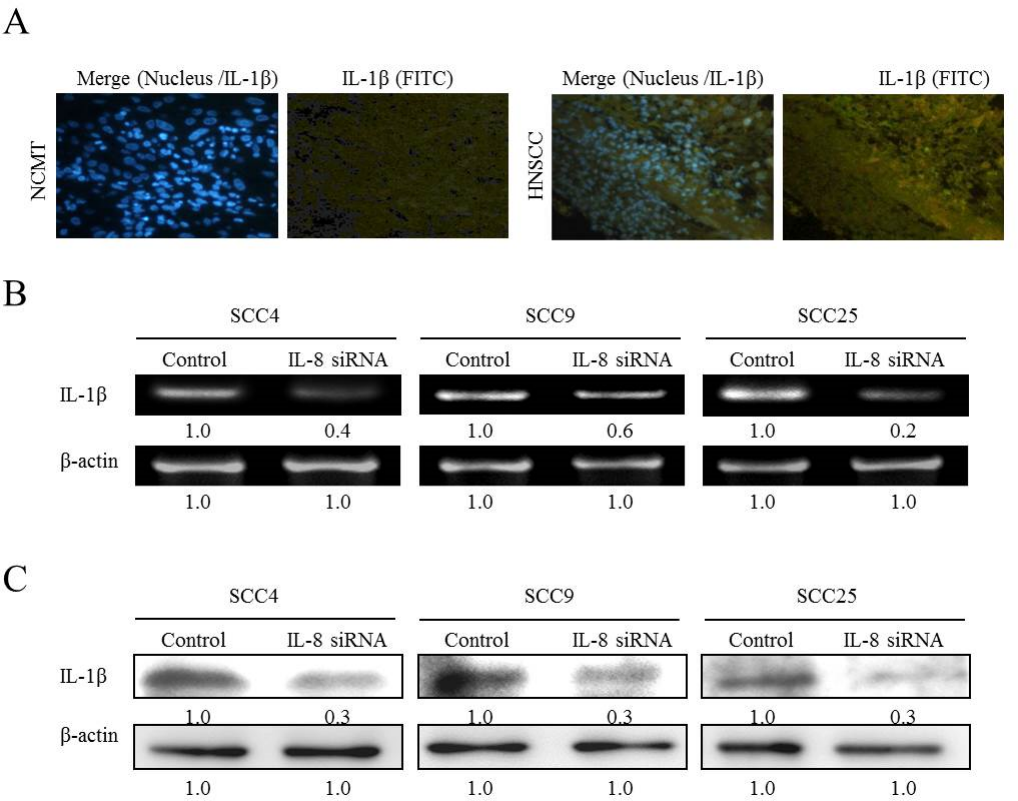
IL-8 siRNA reduces IL-1β expression in HNSCC cells **(A)** Immunohistochemically-stained IL-1β in NCMT and HNSCC, observed under an inverted fluorescent microscope (200× magnification). Knockdown of IL-8 by siRNA reduced expression of IL-1β in three types of HNSCC cell (SCC4, SCC9 and SCC25 cells). Cells were treated with control siRNA and IL-8 siRNA (10 μM) for 6 h, and amount of IL-1β present was determined using RT-PCR **(B)** and western blotting **(C).** Known amounts of β-actin were used as internal controls for sample loading.

To elucidate the role of HNSCC in innate immunity, the inflammatory cytokines (IL-1β, TNF-α and IL-6) that are secreted in response to IL-8, was determined. SCC25 cells were stimulated with IL-8 for 72 h and the cell culture media were collected and screened for IL-1β, TNF-α and IL-6. ELISA experiments revealed that the simultaneous addition of IL-8 (10 and 100 ng/ml) to SCC25 cells promoted the secretion of IL-1β, TNF-α and IL-6 in SCC25 cells (Figure 2C). When HNSCC cells were treated with IL-8, the IL-1β expression was greatly up-regulated relative to the others inflammatory factors by RT-PCR, western blotting and ELISA experiments (Figures 2A-2C). Immunohistochemical staining was carried out to demonstrate the morphological localization of the IL-1β. The immunostaining of IL-1β revealed that it was more highly expressed in the HNSCC cytoplasm than in NCMT (Figure 3A). Replacement of the primary specific antibody with the control (IgG) eliminated staining of all specimens (data not shown). Synthetic siRNA was used to silence IL-1β gene expression by IL-8 RNA interference (IL-8 siRNA). IL-1β gene and protein expressions were then evaluated. Since squamous carcinoma cells (SCC) are present in most patients with head and neck cancer [14], the role of IL-8 in various differentiated SCC cells was examined. SCC4, SCC9 and SCC25 exhibit different differentiations, SCC4 is poorly differentiated, SCC9 is moderately differentiated, and SCC25 is high differentiated. High malignancy is associated with poor differentiation [15, 16]. RT-PCR and western blotting demonstrated that the treatment of HNSCC cells with IL-8 siRNA eliminated the expression of IL-1β by SCC4, SCC9 and SCC25 cells (Figure 3B and 3C) and reduced IL-1β gene expression in three types of HNSCC cell by a factor of 0.4 in SCC4, 0.6 in SCC9 and 0.2 in SCC25 cells (Figure 3B). Treatment of these three types of HNSCC cell with IL-8 siRNA, all of the IL-1β protein levels were reduced by a factor of 0.3 relative to the control siRNA (Figure 3C). However, no obvious finding has been observed on different differentiated SCC by IL-8. These results suggest that IL-8, as the main inflammatory mediator, improved IL-1β expression in HNSCC.

### IL-8 and MAPK signaling pathway in HNSCC

**Figure 4:**
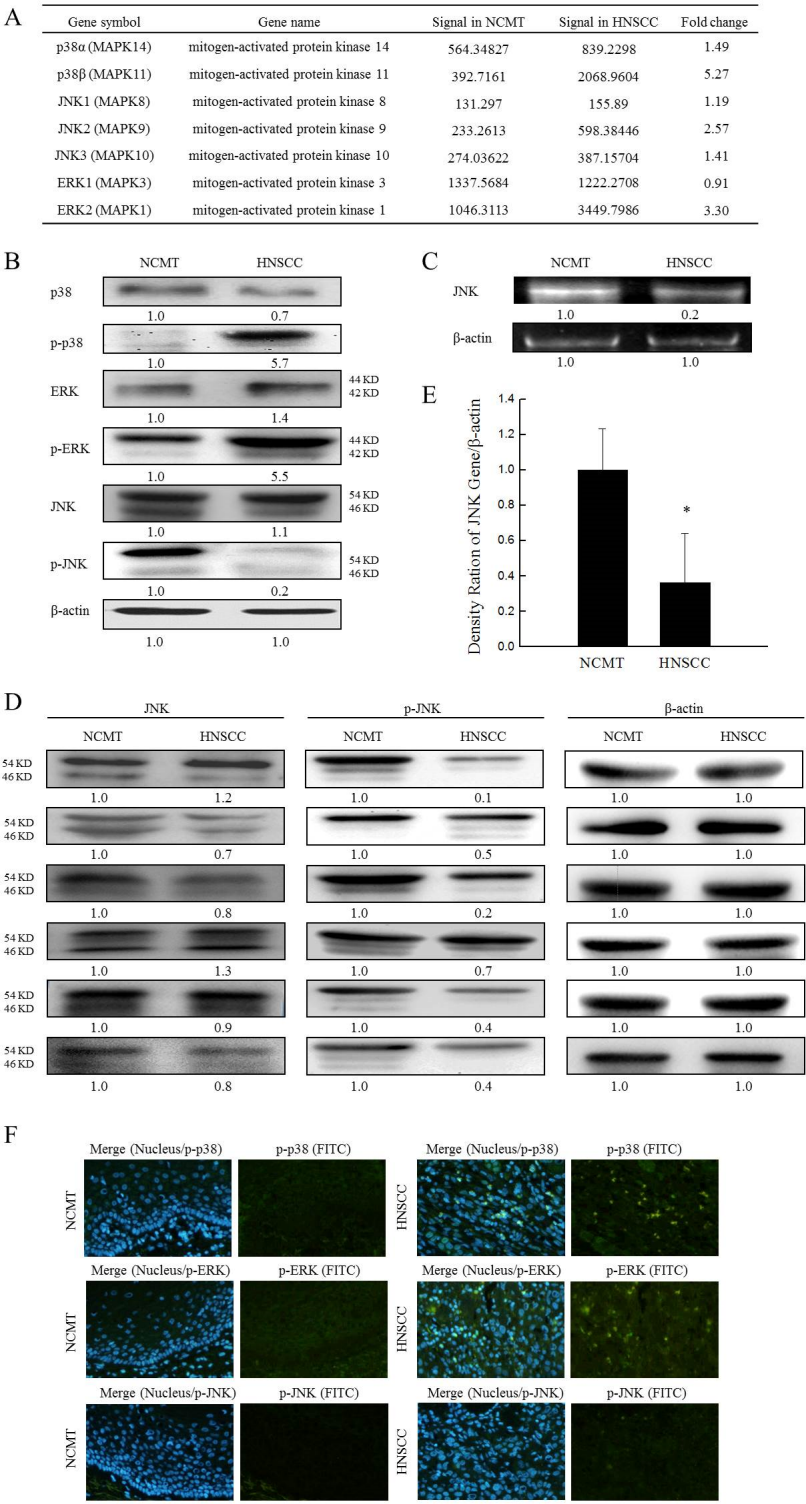
Expression of MAPKs genes in NCMT and HNSCC of human tissue **(A)** A microarray analysis of expressions and fold-change thresholds of p38α, p38β, JNK1, JNK2, JNK3, ERK1 and ERK2 in NCMT and HNSCC of human tissue (patient no. 2). **(B)** Levels of p38 MAPK, p-p38 MAPK, JNK, p-JNK, ERK, p-ERK and β-actin in NCMT and HNSCC of human tissue (patient no. 3) were analyzed by western blotting. **(C)** Gene expression of JNK in NCMT and HNSCC of human tissue were analyzed by RT-PCR. To identify role of JNK in HNSCC, levels of JNK and p-JNK in NCMT and HNSCC tissue (*n* = 6) (patients nos. 1, 4-8) were obtained using western blotting **(D)** and qRT-PCR **(E).** Knows amounts of β-actin were used as internal controls for sample loading. Each value is presented as mean ± SD across three experiments. *p<0.05 implies a significant difference from control cells. **(F)** Immunohistochemically-stained p-p38 MAPK, p-ERK and p-JNK in NCMT and HNSCC, observed under an inverted fluorescent microscope (200× magnification).

MAPK pathway is known as a potential signal transduction pathway that may regulate cell proliferation, survival, growth, inflammation and motility processes, which are critical for carcinogenesis [17]. The role of MAPKs in regulating IL-8-stimulated innate immunity of HNSCC was elucidated. A microarray analysis was performed to elucidate the expression of MAPK genes in HNSCC and NCMT in human tissue. Specifically, a significant 2-fold increase in expression was observed in several genes in the MAPK signaling pathway in HNSCC relative to NCMT, including p38β, ERK2 and JNK2. The increases were by factors of 5.27 for p38β, 2.57 for JNK2 and 3.30 for ERK2 (Figure 4A).

Confirming the microarray data, western blotting demonstrated that p-p38 MAPK and p-ERK exhibited significantly greater and p-JNK had lower expression in HNSCC than in NCMT, and total p38 MAPK, ERK and JNK levels were similarly or less reduced in both types of tissue (Figure 4B). p-p38 MAPK and p-ERK levels were higher (5.7 and 5.5-fold, respectively) in HNSCC than in NCMT, whereas the p-JNK level was a factor of 0.2 lower. These results indicate that the p38 MAPK and ERK ratio was significantly increased (p-p38 MAPK /p38 MAPK = 8.1 and p-ERK/ERK = 3.9) and JNK ratio was significantly decreased (p-JNK/JNK = 0.2) in patients’ tissue. The lower p-JNK protein expressions in HNSCC tissues were consistent with the gene expressions of JNK that were obtained by RT-PCR in comparison with those in NCMT (Figure 4C). The reductions were by a factor 0.2 for JNK in patient tissue with HNSCC, relative to NCMT.

To determine the role of JNK in HNSCC, the total levels of JNK and p-JNK in NCMT and HNSCC tissue (*n* = 6) were evaluated using western blotting and qRT-PCR. Down-regulated p-JNK protein levels in the tissues of six of the HNSCC patients were evaluated by western blotting and compared with those in NCMT, and total JNK levels were found to be similarly or less reduced in both types of tissue (Figure 4D). qRT-PCR analysis was performed to determine the mRNA expression of JNK in six NCMT and HNSCC tissues to verify the results of western blotting (Figure 4E). The mean JNK expression in HNSCC was 0.36 times lower than in NCMT. Experimental data confirmed that the expressions of p-p38 MAPK and p-ERK were greater in the HNSCC cytoplasm than in NCMT, but p-JNK was not expressed in either, according to immunohistochemical analysis (Figure 4F). These results suggest that the expressions of p-p38 MAPK and p-ERK were higher and that of p-JNK was lower in HNSCC patient tissues than in NCMT, suggesting that the abnormal expression of JNK may reflect in HNSCC progression.

### IL-8 regulates MAPK signaling pathway in HNSCC

**Figure 5:**
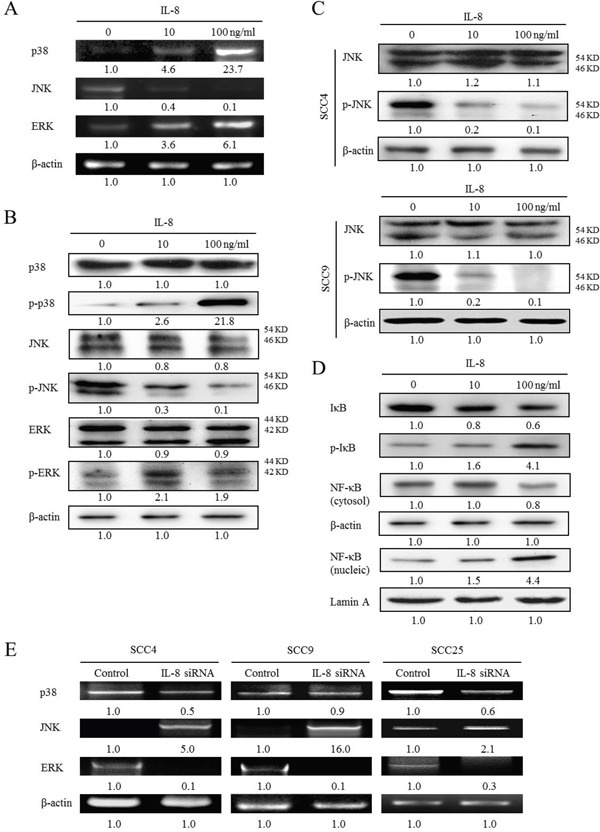
Activation of p38 MAPK/ERK-NF-κB pathway and reduction of JNK expressions in IL-8-stimulated HNSCC cells **(A)** SCC25 cells were stimulated using IL-8 (10 and 100 ng/ml) for 72 h and levels of p38, JNK and ERK were obtained using RT-PCR. **(B)** SCC25 cells were exposed to IL-8 (10 and 100 ng/ml) for 72 h and levels of p38 MAPK, p-p38 MAPK, JNK, p-JNK, ERK and p-ERK, were obtained by western blotting. **(C)** To evaluate IL-8-reduced JNK expression, SCC4 and SCC9 cells were treated with IL-8 (10 and 100 ng/ml) for 72 h, and levels of JNK and p-JNK were evaluated by western blotting. **(D)** To confirm that the p38 MAPK-NF-κB signaling pathway is associated with IL-8, expressions of IκB-α, p-IκB-α, cytosol and nucleic NF-κB in SCC25 cells that were treated with IL-8 (10 and 100 ng/ml) for 72 h were obtained by western blotting. **(E)** Knockdown of IL-8 by siRNA reduced p38 and ERK expressions and increased JNK expression in three types of HNSCC cell (SCC4, SCC9 and SCC25 cells). Cells were treated with control siRNA and IL-8 siRNA (10 μM) for 24 h, and p38 MAPK, JNK and ERK were quantified using RT-PCR. Known amounts of β-actin were used as internal controls for sample loading.

To identify the MAPK-mediated signaling pathway that is associated with IL-8 treatment in HNSCC, the gene expressions of p38 MAPK, JNK and ERK in HNSCC cells that were treated with IL-8 (10 and 100 ng/ml) for 72 h were determined by RT-PCR analysis. Treatment with IL-8 increased p38 MAPK and ERK expressions and reduced JNK expressions in SCC25 cells (Figure 5A). Treatment with IL-8 (100 ng/ml) increased p38 MAPK and ERK mRNA levels 23.7 and 6.1-fold, respectively, and reduced JNK levels. Following the exposure of SCC25 cells to IL-8 (10 and 100 ng/ml) for 72 h, the expressions of p38 MAPK, JNK and ERK were determined by western blotting. IL-8 induced p-p38 MAPK and p-ERK expressions, but reduced p-JNK expression in SCC25 cells (Figure 5B). Treatment with IL-8 (100 ng/ml) increased the p-p38 MAPK and p-ERK protein levels 21.8 and 1.9-fold, respectively, and reduced the p-JNK levels by a factor of 0.1. However, the total p38 MAPK, JNK and ERK protein expressions did not significantly differ between NCMT and HNSCC (Figure 5B). This p38 MAPK, JNK and ERK ratios in HNSCC cells following IL-8 treatment were determined. The ratios of p38 MAPK and ERK; increased (p-p38 MAPK/p38 MAPK = 21.8 and p-ERK/ERK = 2.3) and JNK decreased (p-JNK/JNK = 0.1). Consistent with the reduction of p-JNK expression in SCC25 cells upon treatment with IL-8, differentiated HNSCC cells (SCC4 and SCC9 cells) that exhibited IL-8-induced down-regulation of p-JNK expression were identified. Exposure of SCC4 and SCC9 cells to IL-8 (10 and 100 ng/ml) for 72 h significantly reduced p-JNK expression but did not affect total JNK, as determined by western blotting (Figure 5C). This result indicates that treatment with IL-8 (100 ng/ml) reduced the p-JNK level 0.1-fold in both cells.

A previous study demonstrated that TNF-α increases the production of IL-8 in MHCC-97H cells and p38 MAPK-NF-κB pathways seem to have a critical role in the regulation of IL-8 production [18]. NF-κB is reportedly a major regulator of IL-8 and MCP-1 transcription [19]. The phosphorylation and subsequent degradation of IκB-α is an integral step in the activation of NF-κB [20]. To determine whether the p38 MAPK-NF-κB signaling pathway was associated with IL-8 and affected HNSCC progression, the expressions of IκB-α, p-IκB-α and NF-κB in HNSCC cells that were treated with IL-8 for 72 h were obtained by western blotting (Figure 5D). IL-8 induced the activation of p-IκB-α and nucleic NF-κB, but reduced the expression of IκB-α and cytosol NF-κB in SCC25 cells (Figure 5D). Treatment with IL-8 (100 ng/ml) increased the p-IκB-α and nucleic NF-κB levels 4.1 and 4.4-fold, respectively, and reduced the IκB-α and cytosol NF-κB levels by a factor of 0.6 and 0.8, respectively. This results suggest that IL-8 regulates inflammatory response by modulating the p38 MAPK-NF-κB pathway in HNSCC cells.

IL-8 silencing down-regulated p38 MAPK and ERK expression and up-regulated JNK expression in three types of HNSCC cells (SCC4, SCC9 and SCC25 cells), relative to the control siRNA (Figure 5E). IL-8 siRNA reduced p38 MAPK and ERK gene expressions in three types of HNSCC cell by factors of 0.5 and 0.1 in SCC4, 0.9 and 0.1 in SCC9 and 0.6 and 0.3 in SCC25 cells, respectively. When three types of HNSCC cell were treated with IL-8 siRNA, JNK protein levels increased by a factor of 5.0 in SCC4, 16.0 in SCC9 and 2.1 in SCC25. Experimental data suggest that IL-8 is associated with the up-regulation of p38 MAPK and ERK expressions and the down-regulation of JNK in HNSCC.

### IL-8 is associated with HNSCC migration via regulation of MMPs

**Figure 6:**
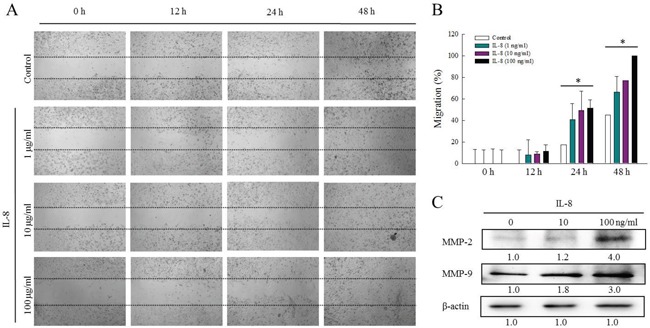
IL-8 is associated with HNSCC migration via regulation of MMPs **(A)** Effect of IL-8 (1, 10 and 100 ng/ml) on proliferation of SCC25 cells was determined using an established wound healing assay, and **(B)** quantitative analysis of migrating cells following IL-8 stimulation was carried out using Image J software. Each value is presented as mean ± SD across three experiments. *p<0.05 implies a significant difference from control cells. **(C)** To determine whether MMPs-mediated HNSCC migration is associated with IL-8 treatment, SCC25 cells were treated with IL-8 (10 and 100 ng/ml) for 72 h, and levels of MMP-2 and MMP-9 were evaluated using western blotting.

A previous study has demonstrated the involvement of ERK in modulating MMP2 and MMP9 and, thereby, cell migration and invasion [21]. To study the effects of IL-8 on HNSCC migration, the effect of IL-8 (1, 10 and 100 ng/ml) on the proliferation in SCC25 cells was determined using an established wound healing assay. Treating SCC25 cells with IL-8 significantly increased cell migration (Figure 6A). Figure 6B presents the quantitative analysis of the migration cells following IL-8 stimulation. The migration of SCC25 cells following exposure to IL-8 increased with its concentration and with time, revealing that IL-8 is involved in HNSCC migration.

To elucidate the MMPs-mediated HNSCC migration that is associated with IL-8 treatment, the expressions of MMP2 and MMP9 in SCC25 cells that were treated with IL-8 were obtained by western blotting (Figure 6C). IL-8 promoted the migration of SCC25 cells and increased MMP-2 and MMP-9 expressions. Treatment with IL-8 (100 ng/ml) increased the MMP2 and MMP9 levels 4.0 and 3.0-fold, respectively. These results suggest that IL-8 promotes HNSCC migration by increasing MMP-2 and MMP-9 expression through the p38 MAPK-NF-κB pathway.

## DISCUSSION

IL-8 has an important role in the pathogenesis of HNSCC, and has been associated with increased tumor growth and metastasis [3]. Our previously obtained results reveal that IL-8 stimulates the proliferation of HNSCC cells. IL-8 not only promotes the formation of HNSCC tissue, but also stimulates NOD pathway activity [22]. These results suggest that IL-8 is important in HNSCC progression via a CXCR1/2-meidated NOD1/RIP2 signaling pathway [22]. IL-8 is believed to act as both an autocrine growth factor in HNSCC cells and as an angiogenic factor [23]. Although the constitutive production of IL-8 appears to be infrequent in most examined cell types, in many of those types, its expression can be induced by particular stimuli, such as GM-CSF, TNF-α, lipopolysaccharide, IL-1β, phorbol 12-myristate 13-acetate, viruses, and double-stranded RNA [24]. Experimental data have revealed that IL-1β, TNF-α, and IL-6 and especially IL-8 levels are higher in HNSCC tissue than in NCMT whereas IL-10 levels are lower, suggesting that IL-8 is the main inflammatory mediator in HNSCC. HNSCC cells were directly treated with IL-8 significantly increasing the expression of IL-1β, TNF-α, and IL-6 and reducing the expression of IL-10. We hypothesize that IL-8 is the main stimulus of inflammatory mediators and co-operates with inflammatory mediators in HNSCC cells. The treatment of HNSCC cells with IL-8 increased the secretion of the IL-1β, TNF-α, and IL-6, and especially increased the secretion of IL-1β. Studies have indicated that IL-1β regulates the ERK pathway of HNSCC progression [25]. Immunohistochemical analysis was utilized herein to demonstrate that IL-1β was expression more in the HNSCC cytoplasm than in NCMT. Knockdown of IL-8 by siRNA reduced the expression of IL-1β in three types of HNSCC cell. Experimental data reveal that IL-1β, TNF-α, and IL-6 were the major inflammatory mediators that were up-regulated by IL-8, and that the up-regulation of IL-1β expression was considerable.

MAPKs have important roles in various cellular activities. For example, ERK phosphorylates various nuclear-binding proteins, including c-Jun/Fos, c-Myc, and E-26-like protein 1, among others, resulting in gene expression, mRNA, and protein synthesis [26]. In mammalian cells, p38 MAPK has a central role in regulating inflammatory responses such as the expression of pro-inflammatory mediators, leukocyte adhesion, chemotaxis, oxidative burst and degranulation [27]. Previous reports have demonstrated that p38 MAPK is not the only signaling route that leads to these cellular responses because ERK, JNK and NF-κB may also be involved [28]. Interaction among these pathways very frequently governs the ultimate biological response to stimulation by inflammatory mediators, which varies with cell type. Transcriptional activation of NF-κB, activation of the JNK pathway, and stabilization of the IL-8 mRNA by the p38 MAPK pathway all regulate IL-8 gene expression [29]. Expression of the IL-8 in neutrophils depends on p38 MAPK following stimulation with TNF-α, GM-CSF, fMLP or LPS, but not with phorbol myristate acetate (PMA) or ionomycin, whereas in endothelial cells, p38 MAPK is not involves in IL-8 production that is induced by IL-1 [10]. Previous reports have demonstrated that IL-8 and p53 expression in HepG2 cells is regulated by the inverse activation of the p38 MAPK, JNK pathways and NF-κB expression [30]. Chronic hepatitis C virus up-regulates cyclooxygenase-2 expression by the IL-8-mediated activation of the ERK/JNK MAPK pathway [31]. Research has established that p38 MAPK signaling is an important posttranscriptional mechanism that underlies the up-regulation of IL-8 mRNA levels that is caused by cigarette smoke extract and acrolein [32]. A previous study demonstrated that the capacity of MEK inhibitor PD98059 to inhibit IL-6 and IL-8 secretion upon stimulation with lysophospholipids is related to its inhibition of ERK kinase activation in immature or maturing dendritic cells but not in mature dendritic cells [33]. However, the IL-8-mediated MAPK signaling pathway in HNSCC has not been clearly elucidated. Our results concerning IL-8-induced p38 MAPK and ERK expression in HNSCC are consistent with these previous findings [29–33]. Higher p38 MAPK and ERK levels were identified herein in HNSCC tissue than in NCMT. Moreover, this work demonstrated that the stimulation of HNSCC cells with IL-8 induces the p-p38 MAPK and p-ERK expression. Several lines of evidence suggest that the ERK MAPK pathway, but not the JNK pathway or the p38 MAPK pathway, is a major regulator of cell proliferation in colorectal cancer [34]. Previous studies have established that IL-8 in human neutrophils activates p38 MAPK and ERK but not JNK, suggesting that IL-8 selectively activates MAPK pathways in neutrophils [35]. In this study, lower gene and protein expressions of JNK were detected in HNSCC than in NCMT in human specimens. Treating three types of HNSCC cell with IL-8 siRNA significantly reduced the expression of p38 MAPK and ERK, but increased that of JNK. Experimental data suggest that IL-8 is associated with the up-regulation of p38 MAPK and ERK expressions and the down-regulation of JNK expression in HNSCC. IL-8 induced the activation of p-IκB-α and nucleic NF-κB, but reduced IκB-α and cytosol NF-κB expression in SCC25 cells. These results suggest that IL-8 regulates inflammatory response by modulating the p38 MAPK-NF-κB pathway in HNSCC cells. A previous study demonstrated that IL-8 induces proliferation and migration of endothelial cells and the production and angiogenesis of MMP-2 [36]. However, the mechanism of IL-8-modulated MMPs that is associated with HNSCC migration not been clearly identified. In this investigation, the potential for cell migration potential was assessed using an established wound healing assay, which revealed that IL-8 promotes the proliferation and migration of SCC25 cells. These experimental results reveal that the exposure of SCC25 cells to IL-8 increased MMP2 and MMP9 expression. These results suggest that IL-8 promotes the HNSCC migration by increasing MMP-2 and MMP-9 expression through the p38 MAPK-NF-κB pathway.

**Figure 7:**
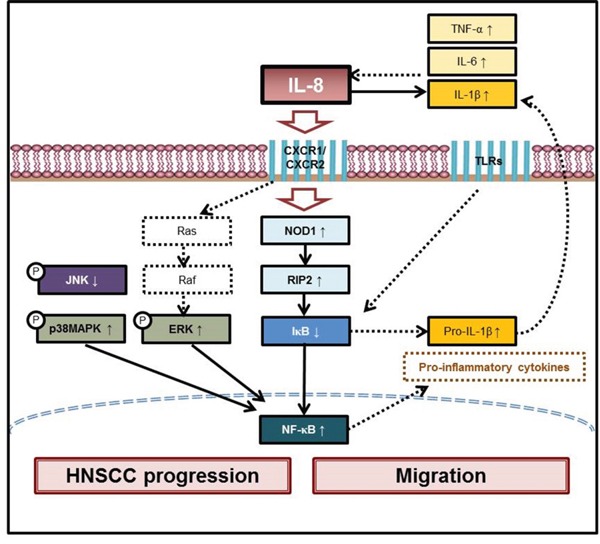
Scheme of IL-8 promotes inflammatory mediators in HNSCC progression and migration Our previous report established that IL-8 promotes HNSCC progression along CXCR1/2-mediated NOD1/RIP2 signaling pathway. This study reveals that IL-8 promotes inflammatory mediators and stimulates both activation of p38 MAPK/ERK-NF-κB pathway and reduction of JNK level in HNSCC progression and migration. Two instances of IL-8-mediated mechanical signaling in HNSCC cells are schematically depicted. Results in this figure can be utilized to develop biomarkers and targets in HNSCC and to provide a basis for early diagnosis and treatment.

A commonly used chemotherapeutic drug, paclitaxel, has been shown to have potentially positive effects in the treatment of ovarian cancer, and this finding is believed to be related to, in part, the observed activation of p38 MAPK in cells upon with paclitaxel [37]. Herein, IL-8 is characterized as a major stimulus of inflammatory mediators as it up-regulated the inflammatory mediators in HNSCC (Figure 7). This study investigates IL-8 expression in surgical specimens of HNSCC and its relationship with clinicopathological factors. HNSCC cells were stimulated using IL-8 and the p38 MAPK, JNK and ERK pathways were elucidated. Relevant findings constitute the first evidence that IL-8 increases p38 MAPK and ERK expressions and reduces JNK expression in HNSCC. This information can be exploited to develop biomarkers and targets in HNSCC and to provide a basis for early diagnosis and treatment. Further validating investigations may have to be performed using a larger cohort to compare cases of HNSCC with healthy control groups to provide a greater statistical significance. More research into the effects of IL-8 and MAPK signaling pathways that are associated with inflammation in the near future would establish their usefulness as biomarkers for HNSCC.

## MATERIALS AND METHODS

### Patients and specimens

The Department of Otolaryngology-Head and Neck Surgery, Kaohsiung Medical University Hospital, Kaohsiung Medical University, Kaohsiung, Taiwan, approved this investigation. A retrospective study of SCC specimens that had been obtained from eight patients who underwent surgical resection at the Kaohsiung Medical University Hospital in 2013 was conducted. Of the eight patients with SCC, six were men and two were women. The mean age of the patients was 53 years (age range, 43-75) (Table 1). All patients had recently been diagnosed with a primary disease, and had not received any prior treatment in the form of chemotherapy, radiotherapy or alternative remedies. All tissue samples were fresh frozen in liquid nitrogen until RNA and protein purification was carried out and microarray experiments were performed; they collected with patient consent under the approval of the institutional review boards of all participating institutions.

**Table 1:** Summary of HNSCC patients

Patient No.	Age (y)	Sex	Site	P-stage	Staging
1	50	M	L tongue	pT1N0M0	I
2	55	M	L tongue	pT2N0M0	II
3	75	F	R tongue	pT1N1M0	III
4	43	M	L tongue	pT2N2bM0	IV
5	47	M	L tongue	pT2N2bM0	IV
6	53	M	L tongue	pT2N2cM0	IV
7	51	M	L tongue	pT2N2bM0	IV
8	52	F	R tongue	pT4aN0M0	IV

### Cell culture

HNSCC cells (poorly differentiated SCC4 cells, moderately differentiated SCC9 cells and well differentiated SCC25 cells) were obtained from the American Type Culture Collection (Rockville, MD). HNSCC cells were maintained in Dulbecco's modified Eagle medium (DMEM)/F12 that had been supplemented with 0.4 μg/ml hydrocortisone (GIBCO, Grand Island, NY) and 10% fetal bovine serum (FBS) (Hazelton Product, Denver, PA, USA). All cells were incubated at 37°C in a humidified atmosphere of 5% CO_2_ in air.

### Microarray

To profile the expression of NCMT and HNSCC, total tissue RNA was extracted using Trizol reagent (Invitrogen, Carlsbad, CA, USA), and then the RNeasy Mini Kit (Qiagen, Hilden, Germany) was used. The isolated RNA was quantified at OD 260 nm and qualitatively analyzed using a bioanalyzer (Agilent Technology, USA). A 0.2 μg mass of total RNA was amplified using a Low-Input Quick-Amp Labeling Kit (Agilent Technologies, USA) and labeled with Cy3 (CyDye, Agilent Technologies, USA) during an *in vitro* transcription process. A 0.6 μg mass of Cy3-labled cRNA was fragmented to a mean size of approximately 50-100 nucleotides by incubation with a fragmentation buffer at 60°C for 30 minutes. Similarly fragmented labeled cRNA is then pooled and hybridized using an Agilent Sure Print G3 Human V2 GE 8 × 60 K Microarray (Agilent Technologies, USA) at 65°C for 17 h. After the microarrays are washed and dried by blowing with a nitrogen gun, they are scanned using an Agilent microarray scanner (Agilent Technologies, USA) at 535 nm for Cy3. Scanned images are analyzed using Feature Extraction 10.5.1.1 software (Agilent Technologies, USA) for image analysis and normalization to quantify the signal and background intensity for each feature (Welgene Biotech. Co., Taipei, Taiwan).

### Western blot analysis

For the extraction of protein from NCMT and HNSCC tissue, frozen tissue was homogenized in lysis buffer (50 mM HEPES, pH 7.5, 150 mM NaCl, 10% glycerol, 1% Triton X-100, 1mM EDTA, 1 mM EGTA, 50 mM NaF, 1 mM sodium orthovanadate, 30 mM *p*-nitrophenyl phosphate, 10 mM sodium pyrophosphate, 1 mM phenylmethylsulfonyl fluoride, 10 μg/ml aprotinin, and 10 μg/ml leupeptin). Supernatants were collected and analyzed. To extract cellular protein, HNSCC cells (1 × 10^5^ cells/ml) were treated with IL-8 (10 and 100 ng/ml) for 72 h, and then washed with cold PBS before immediately being lysed using lysis buffer.

Tissue extracts and cell lysates were prepared and subjected to western blotting with antibodies against IL-1α (sc-271618), IL-1β (sc-7884), IL-6 (SC-130326), IL-8 (sc-8427), IL-10 (sc-8438), TNF-α (sc-33639), p38α/β (sc-7972), p-p38 (sc-166182), ERK1/2 (sc-135900), p-ERK1/2 (sc-81492), JNK (sc-7345), p-JNK (sc-6254), IκB-α (sc-373893), p-IκB-α (sc-8404) and NF-κB (sc-8008), Lamin A (sc-56137), MMP-2 (sc-13595), MMP-9 (sc-21733) and β-actin (sc-47778) (Santa Cruz, CA). Proteins were detected using enhanced chemiluminescence (ECL) (Amersham Biosciences, Buckinghamshire, UK) and exposed to a luminescent image analyzer (Immobilon-P, Merck Millipore, Darmstadt, Germany). The relative expression of each was obtained by densitometry using Image J software and normalized relative to β-actin. To analyze the level of nuclear NF-κB, nuclear and cytoplasmic proteins were separated using an NE-PER™ Nuclear and Cytoplasmic Extraction Reagents kit (Thermo Scientific™, Waltham, MA, USA). Lamin A was adopted as an internal control for sample loading.

### Reverse transcription-polymerase chain reaction (RT-PCR) analysis

For NCMT and HNSCC tissue RNA isolation, RNA was extracted using the Total RNA Miniprep Purification Kit (GeneMark). For cellular RNA extraction, HNSCC cells (1 × 10^5^ cells/ml) were treated with IL-8 (10 and 100 ng/ml) for 72 h, and the RNA was isolated using the Trizol reagent (Invitrogen, Carlsbad, CA, USA).

Single-stranded cDNA was transcribed by priming with oligo-dT with SAMscript reverse transcriptase (GeneMark, Taipei, Taiwan). PCR amplification of the cDNA was performed in a reaction mixture that contained *Taq* polymerase (GeneMark, Taipei, Taiwan). The primers were as follows; IL-1α (107 bp), 5′-GACGCACTTGTAGCCACGTA-3′ and 5′- ACCGCCAATGAAATGACTCC -3′; IL-1β (65 bp), 5′-ACGAATCTCCGACCACCACT-3′ and 5′- CCA TGGCCACAACAACTGAC-3′; IL-6 (159 bp), 5′- GTG TGAAAGCAGCAAAGAGGC-3′ and 5′- CTGGAGGT ACTCTAGGTATAC -3′; IL-10 (266 bp), 5′-AAGCTGAGAACCAAGACCCAGACATCAAGGCG-3′ and 5′-AGCTATCCCAGAGCCCCAGATCCGATTTTGG-3′; TNF-α (224 bp), 5′-TTCCTCACTCACACC ATCAGCC -3′ and 5′-TGCCCAGATTCAGCAAAGTCC-3′; p38 (205 bp), 5′-ATGAAGCTCTCCAACACCCG-3′ and 5′-GCACCTAAAGGAGAGGGCTG-3′; JNK (159 bp), 5′-CTGAAGCAGAAGCTCCACCA-3′ and 5′-CACCTA AAGGAGAGGGCTG-3′; ERK (150 bp), 5′-GCTCAC CCTTACCTGGAACA-3′ and 5′-GGACC AGATCCA AAAGGACA-3′; and β-actin (295 bp), 5′-TCACC CACACTGTGCCCATCTACGA-3′ and 5′-CAGCGGAACCGCTCATTGCCAATCG-3′. The amplified RT-PCR products were electrophoresed on a 2% agarose gel, visualized by ethidium bromide staining and photographed under ultraviolet light. The obtained bands were visualized using image J software and the levels were normalized to β-actin expression.

### Enzyme-linked immunosorbent assay (ELISA)

SCC25 cells were seeded at a density of 2 × 10^5^ cells/mL into a 24-well plate that contained medium with 10% FBS, and cultured overnight. The medium was then exchanged, and the cells were cultured for another 48 h. The culture media were then collected and centrifuged at 1,500 rpm for 5 min to remove particles, and the supernatants were frozen at −80°C until use with the ELISA kit. The concentrations of IL-1β, IL-6 and TNF-α were measured using a BD OptEIA ELISA kit (BD PharMingen, San Diego, CA), following the manufacturer's instructions. To elucidate the effect of production of inflammatory mediators in SCC25 cells, these cells were stimulated using IL-8 (10 and 100 ng/ml) and cultured them for 72 h; the concentrations of IL-1β, IL-6 and TNF-α were measured using the aforementioned methods.

### Immunohistochemical staining

Paraffin-embedded biopsies were carried out using anti-IL-1β (sc-7884), p-p38 (sc-166182), p-ERK1/2 (sc-81492) and p-JNK (sc-6254); immunohistochemical analysis followed. The primary antibody were diluted 1:400 in phosphate buffered saline and added to the tissue sections and incubated overnight in a moist chamber at 4°C; then corresponding secondary antibodies with FITC. Controls were established by incubating slides with an IgG isotype control rather than primary antibodies. The section was also stained with Hoechst 33342 to study the nuclear morphology. The specific protein expressions and cell nuclei were investigated under a fluorescence microscope (Nikon, TE2000-U, Japan).

### Small interfering RNA (siRNA) transfection

Human IL-8 (sc-39631) and non-silencing (sc-37007) small interfering RNAs (IL-8 siRNA) were obtained from Santa Cruz Biotechnology (Santa Cruz, CA, USA).

The transfection of siRNA was performed with Lipofectamine RNAiMAX transfection reagent (Invitrogen) following the manufacturer's protocol. Briefly, one day prior to transfection, cells were seeded in a 6-well plate that contained antibiotic-free serum. At the time of transfection, cell confluence was 50% and the medium was replaced with antibiotic-free growth medium. IL-8 and negative control siRNA were diluted in 500 μl of DMEM/F12, and then 5 μl Lipofectamine RNAi MAX reagent was added; it was then left to stand at room temperature for 20 min. The diluted RNAi-Lipofectamine RNAiMAX mixture was added to the cells. The cells were incubated at 37°C in a CO_2_ incubator for 6 h. After 6 h, the supernatants were removed and the medium was replaced with fresh medium. After 24 h of incubation, the cells were harvested, and the expression of the transfected gene was evaluated by RT-PCR and western blotting.

### Quantitative real-time PCR (qRT-PCR)

For NCMT and HNSCC tissue RNA isolation, total RNA was extracted using a Total RNA Miniprep Purification kit (GeneMark). The primers were as follows; JNK, 5′-' CGCCCAATACGACCAAATCAGA-3′ and 5′-ATGTTGCAACCGGGAAGGAA-3′; and β-actin, 5′-TGTCCACCTTCCAGCAGATGT-3′ and 5′-AGTC CGCCTAGAAGCATTTGC-3′. The amplification reactions were performed in 20 μl of a mixture, using SYBR Green Supermix (Bio-Rad Laboratories). The cycles were set at 95°C for 10 min for preheating, followed by 30 cycles at 95°C for 15 s, at 56°C for 30 s and at 72°C for 30 s. All PCRs were normalized to the internal control β-actin mRNA.

### Wound healing assay

SCC25 cells were grown as a monolayer on culture plates in the presence and the absence of IL-8 (1, 10 and 100 ng/ml). The monolayer was scratched using a sterile pipette tip, and then washed with PBS or serum free media to remove traces of growth factors. Cell migration across the wound was observed using a microscope (Nikon, TE2000-U, Japan) and recorded photographically at 0, 12, 24 and 48 h. Percentage migration was quantified using Image J software as (100 – area of scratch at specified concentration of IL-8/area of scratch of control*100)

### Statistical analysis

The results are expressed as mean ± standard deviation (SD). Student's *t*-test was used to analyze the assays. A *p* value of 0.05 was regarded as significant. The data were analyzed and plotted using software (SigmaPlot Version 8.0 and SigmaStat Version 2.03, Chicago, IL).
